# Electroacupuncture for migraine protocol for a systematic review of controlled trials

**DOI:** 10.1097/MD.0000000000009999

**Published:** 2018-04-27

**Authors:** Hongmin Chu, Jihye Seo, Cheolhyun Kim, Yeonju Moon, Dae Gill Kang, Ho Sub Lee, Kang-Keyng Sung, Sangkwan Lee

**Affiliations:** a Department of Internal Medicine, College of Korean Medicine, Wonkwang University, Iksan, Jeonbuk, Republic of Korea; b Clinical Trial Center, Wonkwang University Gwangju Hospital, Gwangju, Republic of Korea; c Hanbang Cardio-Renal Syndrome Research Center, College of Oriental Medicine, Wonkwang University, Iksan, Jeonbuk, Republic of Korea; d Internal Medicine & Neuroscience, Jangheung Integrative Medical Hospital, Wonkwang University, Jangheung, Jeonnam, Republic of Korea.

**Keywords:** electroacupuncture, migraine, protocol, systematic review

## Abstract

**Background and Objective:**

A migraine is one of the primary headache disorders. Acupuncture has been widely used to treat migraine. Furthermore, electroacupuncture (EA) treatment has been also used to treat migraine. However, there has been no systematic review by assessing efficacy and safety of EA on migraine. This protocol is developed to conduct a systematic review and meta-analysis to evaluate the evidences related to the effectiveness and safety of EA on migraine.

**Methods and analysis:**

This protocol follows the guideline according to the Preferred Reporting Items for Systematic reviews and Meta-Analysis Protocol and registered on the International Prospective Register of Systematic Reviews (PROSPERO). The following seven databases will be searched from their inception to September 2016: Medline, the Cochrane Central Register of Controlled Trials (CENTRAL), EMBASE, OASIS, the Korean Traditional Knowledge Portal, the Korean Medical Database and the China National Knowledge Infrastructure (CNKI). This Systemic review will include only the randomized controlled clinical trials (RCTs) of acupuncture therapy on migraine. We will perform data extraction, study selection, assessment with risk of bias and data analysis. The primary outcomes of this study are headache pain intensity and the total treatment effective rate. And this protocol study for systematic reviews, the approval of IRB was not required.

**Ethics and dissemination:**

This systematic review will not need ethical approval, because it doesn’t involve human beings. We will publish this systematic review electronically in a peer-reviewed journal. This systematic review will give healthcare practitioners good practical guide and information for treating migraine.

**Systematic review registration:**

PROSPEROCRD42018085099

## 1 Introduction

A migraine is a primary headache disorder that most often begins at puberty and affects those aged between 35 and 45 years. Migraine is caused by the activation of a mechanism deep in the brain that leads to the release of pain-producing inflammatory substances around the nerves and blood vessels of the head.^[1]^ In recent population-based studies in the United States, 14.2% of US adults reported having migraine or severe headache in the previous 3 months.^[2]^ According to ICHD-III (International Classification of Headache Disorders, 3rd edition beta version),^[3]^ migraine has two major subtypes, migraine without or with aura. Migraine without aura is a clinical syndrome characterized by headache with specific features and associated symptoms. Migraine with aura is primarily characterized by the transient focal neurological symptoms that usually precede or sometimes accompany the headache. Acupuncture has been a widely used method to treat migraine in Asia and Western countries.^[4,5]^ In addition, electroacupuncture (EA) has been also used to treat migraine.^[6]^ EA is a modern way of administering acupuncture^[7]^ defined as the passage of a pulsed electric current through the body tissue via one (or more) pairs of acupuncture needles for therapeutic purpose. EA is known to be effective for persistent pain,^[8]^ neuropathic pain,^[9]^ and analgesia.^[10]^ Several systematic reviews of EA have been published.^[11–13]^ However, to our knowledge, there has been no systematic review on the effectiveness of EA on migraine. Therefore, we propose the current protocol to perform the systematic review by assessing the evidences related to the effectiveness of EA on migraine.

## 2 Methods and analysis

This protocol follows the guidelines according to the preferred reporting items for systematic reviews and meta-analysis protocol (PRISMA-P).^[14]^ We will use the Preferred Reporting Items for Systematic Reviews and Meta-Analyses (PRISMA) guidelines and the Cochrane Handbook for Systematic Reviews of Interventions. The protocol for this systematic review was registered on PROSPERO with registration number: PROSPEROCRD42018085099.

### 2.1 Data sources and search methods

This study will electronically search Medline, EMBASE, and the Cochrane Central Register of Controlled Trials (CENTRAL). We will also search the following Korean and Chinese databases: four Korean medical databases (OASIS, the Korean Traditional Knowledge Portal, the Korean Medical Database, and DBPIA), one Chinese database (the China National Knowledge Infrastructure). All databases will be searched from their inception to September 2016.

The searching terms to be used will include migraine Disorders, headache, migraine attack and episodic migraine, cephalalgi. We will also search the terms electroacupuncture as well as acupuncture, acupuncture therapy, acupuncture, acupuncture analgesia and acupuncture points for extensive searches. The example search strategy in Table 1 will be used for Medline. This search strategy will be modified and used for the other databases.

**Table 1:**
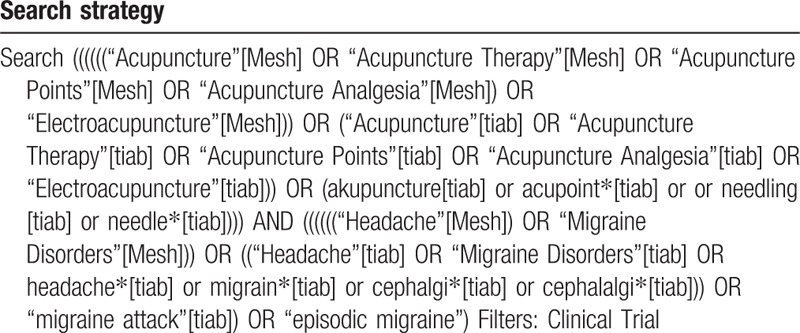
Search strategy will be used for Medline.

### 2.2 Inclusion criteria

#### 2.2.1 Types of participants and interventions

Patients with diagnosed migraine will be included. The diagnostic criteria are the International Classification of Headache Disorders or its equivalent. We will include participants of either sex and of any age. Interventions to be reviewed are EA sole treatment or as a combination with other treatments to treat the migraine. In case of the combination with other treatments, only the study that the control group received the same treatment as the intervention group will be included.

#### 2.2.2 Types of studies

This systematic review will include randomized controlled clinical trials (RCTs) including quasi-randomized controlled trials (quasi-RCTs). We will exclude any types of studies such as controlled (non-randomized) clinical trials (CCTs), case series, and case reports. Considering the language restriction of our researchers, the included studies will be limited to the literature written in English, Korean or Chinese. Articles whose full text can be obtained will be included.

#### 2.2.3 Types of outcomes

The primary outcomes will be the therapeutic effects of treatment on the Migraine. This will be headache pain intensity and the total treatment effective rate. Headache pain intensity may be indicated on the visual analogue scale or NRS. The total treatment effective rate is the number of patients with improvement in the number of migraine attacks or of the migraine symptoms. This will show the effectiveness.

Secondary outcomes will include the following measures:

1. Impact of migraine-related symptoms (headache frequency, headache duration time) as measured with validated questionnaires
2. Migraine-associated symptoms (nausea, photophobia, phonophobia)
3. Quality of life: evaluated by general or migraine-specific scales
4. Adverse events

#### 2.2.4 Study selection

After searching process, the results will be exported to the Endnote referencing software and duplicate studies will be removed using this software. The selection process will be performed by two authors independently. Initially, we will screen and evaluation the titles and abstracts of studies, and select those likely to be of relevance to our systematic review. In the second stage of selection, we will assess the full-text of the studies and confirm the eligibility for our review. When there are any disagreements, we will resolve the disagreements by discussion. Using the PRISMA-compliant flow chart (http://www.prisma-statement.org), this study screening and selection process will be documented and summarized. In this flowchart, the reasons for excluding studies will be provided.

#### 2.2.5 Data extraction and assessment with risk of bias

Data extraction and quality assessment of studies will be performed by two independent authors. In this process, the results will be cross-checked. Any disagreement between the results derived from two authors will be resolved with a discussion. If the disagreement cannot be resolved with a discussion, it will be resolved by the arbiter.

To perform the data extraction and quality assessment of the RCTs, we will use a data extraction form (Excel). The extraction form will be designed by all the authors in consensus. The data to be extracted will include the first author, year of publication, patient characteristics, intervention and comparison details, sample size and dropouts, outcomes and adverse events.

The quality assessment will be performed using the “Risk of bias”’ tool from the Cochrane Handbook V.5.1.0. The “Risk of bias” tool includes random sequence generation, allocation concealment, blinding of the participants and personnel, blinding of the outcome assessments, incomplete outcome data, selective reporting, and other sources of bias.

#### 2.2.6 Data analysis

We will do data analysis to compare the outcomes between the intervention and control groups. Data analysis will be performed with the values that measured at the end of the treatment period. When quantitative synthesis, we will analyze a single measurement of each outcome in each participant. Each participant's outcome measurement will be counted only once. The data synthesis will be performed with Rev Man V.5.2.7 (the Cochrane Collaboration's software program Review Manager) for Windows.

For dichotomous data, we will present the outcomes as relative risks (RRs) with 95% CIs. For continuous data, we will calculate the effect size of the interventions using the mean differences (MDs) with 95% CIs. If the trials present the outcome values on different scales, we will use the standard mean difference (SMD) with 95% CIs. We will calculate the data for the meta-analysis using fixed or random effects.

For studies that contain insufficient information, we will contact the authors to request the insufficient data whenever possible. If data synthesis is impossible, we will use narrative analysis.

### 2.3 Assessment of heterogeneity

We will assess the heterogeneity by visually inspecting the forest plots and calculating the *I*^2^ statistic. *I*^2^>50 will be considered to indicate high heterogeneity. In cases of high heterogeneity, we will perform and present subgroup analyses to explore the possible causes.

### 2.4 Assessment of reporting biases

To assess the reporting biases, we will assess the publication bias with a funnel plot when a sufficient number of included studies (at least 10 trials) are available. We will also attempt to determine the possible reasons for any asymmetries in results, such as a selective outcome reporting bias, small-study effects, a poor methodology, or true heterogeneities in the included studies.

### 2.5 Subgroup analysis

In cases of high heterogeneity, we will perform a subgroup analysis to explore and assess the heterogeneity. We will conduct subgroup analyses according to the different combinations of cupping therapies with treatments such as blood-letting, or to the other factors affecting the outcomes.

### 2.6 Quality of evidence

To evaluate the quality of our review's evidence, we will use the Grading of Recommendations Assessment, Development and Evaluation (GRADE).

## 3 Discussion

EA is a CAM therapy commonly used to treat migraine.^[6]^ Some studies have suggested that EA is an effective treatment for post-stroke spasticity,^[11]^ knee osteoarthritis,^[12]^ tinnitus, ^[13]^ and Obesity.^[15]^ However, there has been no systematic review showing the evidences of the effectiveness and safety of EA on migraine. We have presented a protocol for a systematic review of EA for migraine treatment. However, this protocol may have some limitations. First, the inclusion is limited to the studies of Chinese, English, and Korean. Second, different dosage of herbal medicine, age of patients and small sample size of trials may lead to some bias. We will publish this systematic review of EA in a peer-reviewed journal. If there are the critical changes of this protocol, we will write the changes in the review. This study will form the basis to conduct additional research and provide evidence for the effectiveness of EA for migraine treatment.

## 4 Authors’ contributions

HC wrote and registered this protocol and drafted the study manuscript, JS developed the search methods and performed data analysis and CK examined the inclusion criteria in clinical practice. SL revised the protocol. All authors have read and approved the final manuscript.
